# One-pot tandem cyclization of enantiopure asymmetric *cis*-2,5-disubstituted pyrrolidines: Facile access to chiral 10-heteroazatriquinanes

**DOI:** 10.3762/bjoc.9.32

**Published:** 2013-02-07

**Authors:** Ping-An Wang, Sheng-Yong Zhang, Henri B Kagan

**Affiliations:** 1 Department of Medicinal Chemistry, School of Pharmacy, The Fourth Military Medical University, Changle Xilu 17, Xi-An, 710032, P. R. China; 2 Institut de Chimie Moléculaire et des Matériaux d’Orsay (ICMMO-UMR 8182, CNRS), Laboratoire de Catalyse Moléculaire, Université Paris-Sud, 15 rue Georges Clemenceau, 91405 Orsay Cedex, France

**Keywords:** *cis*-2,5-disubstituted pyrrolidine, 10-heteroazatriquinane, tandem cyclization, X-ray single-crystal diffraction analysis

## Abstract

A series of chiral 10-heteroazatriquinanes were synthesized from enantiopure asymmetric *cis*-2,5-disubstituted pyrrolidines through a one-pot tandem cyclization procedure. The structures and configurations of these new chiral 10-heteroazatriquinanes are confirmed by X-ray single-crystal diffraction analysis.

## Introduction

**Figure 1:**
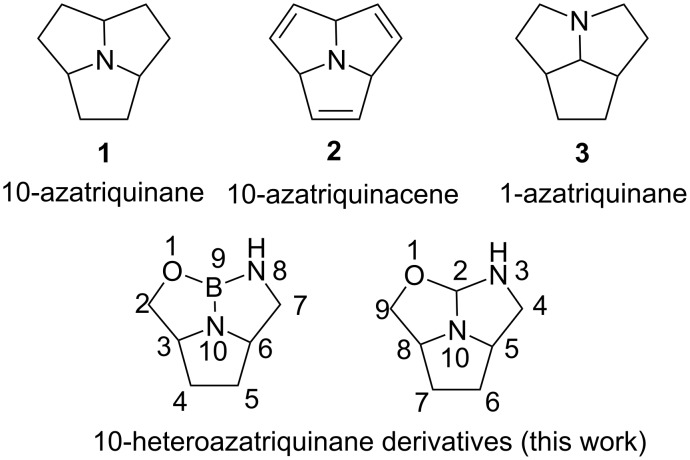
The structures of azatriquinanes and azatriquinacene.

The azatriquinane derivatives are an important substance class in organic chemistry containing nitrogen and three fused five-membered rings [1–3]. Due to the unique rigid bowl-shaped structure with one noninversible electron lone pair at the bottom of the central nitrogen (“*centro*-N”) atom, 10-azatriquinane analogues are used as efficient chelation reagents of metal cations [4–5]. Mascal and colleagues [6] described the first synthesis of 10-azatriquinane (**1**) from dimethyl 3,3'-(1*H*-pyrrole-2,5-diyl)dipropanoic acid in five steps, and the reactivity of **1** was also investigated. 10-azatriquinane (**1**) is very active because of its high basicity. The X-ray structure of **1**·HBF_4_ revealed that the *centro*-N is pyramidalized. 10-azatriquinacene (**2**), an unsaturated analogue of **1**, was attractive due to its high proton affinity [7]. Recently, Mascal [8] and colleagues have developed a series of 10-azatriquinanes as a *C*_3_*_v_*-symmetric platform for tripodal metal complexes and calixiform scaffolds (Figure 1).

**Figure 2:**
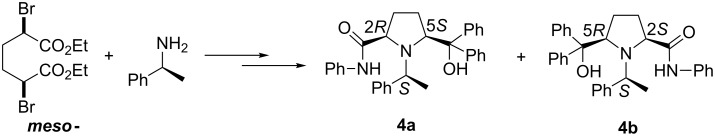
The synthesis of **4** (*previous work*).

**Scheme 1:**
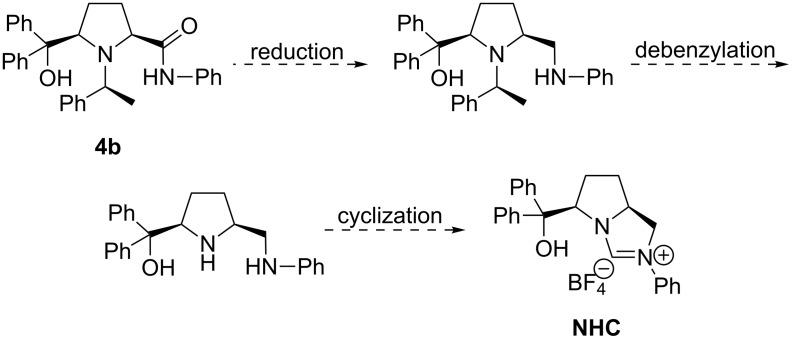
The designed synthetic route to a hydroxy NHC from **4b**.

Previously, we established a facile access to enantiopure asymmetric *cis*-2,5-disustituted pyrrolidines **4** from commercially available starting materials diethyl *meso*-2,5-dibromoadipate and (*S*)-(−)-1-phenylethylamine (Figure 2) [9]. The preparations of compounds **5a**, **5b**, **6a** and **6b** were also reported in [9], but our previously published structures for compounds **5a** and **5b** were not completely correct, because the B–N dative bonds were missing. The aim of the following procedures was to obtain several novel bifunctional *N*-hetereocyclic carbenes (NHCs) [10–14] from compounds **4** through three steps, including reduction, debenzylation and cyclization (Scheme 1), but this failed. However, we have found a novel one-pot tandem cyclization of these enantiopure asymmetric *cis*-2,5-disubstituted pyrrolidines to produce chiral trisubstituted 10-heteroazatriquinane derivatives. To the best of our knowledge, there is very little research on the synthesis of 10-heteroazatriquinanes, and the chiral 10-heteroazatriquinanes are unknown up to now.

## Results and Discussion

**Scheme 2:**
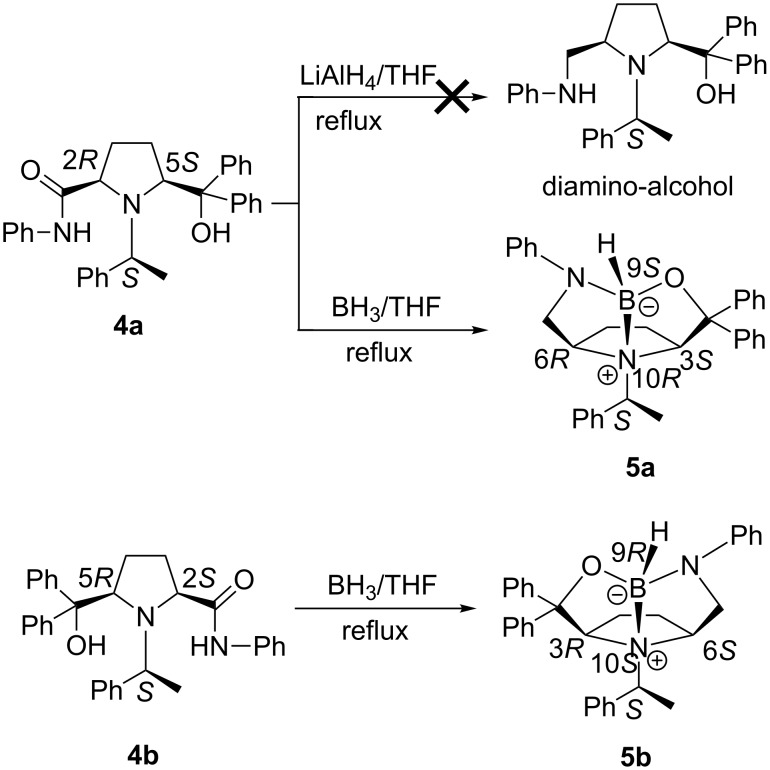
The reduction of amides **4** by LiAlH_4_ and BH_3_·THF.

**Figure 3:**
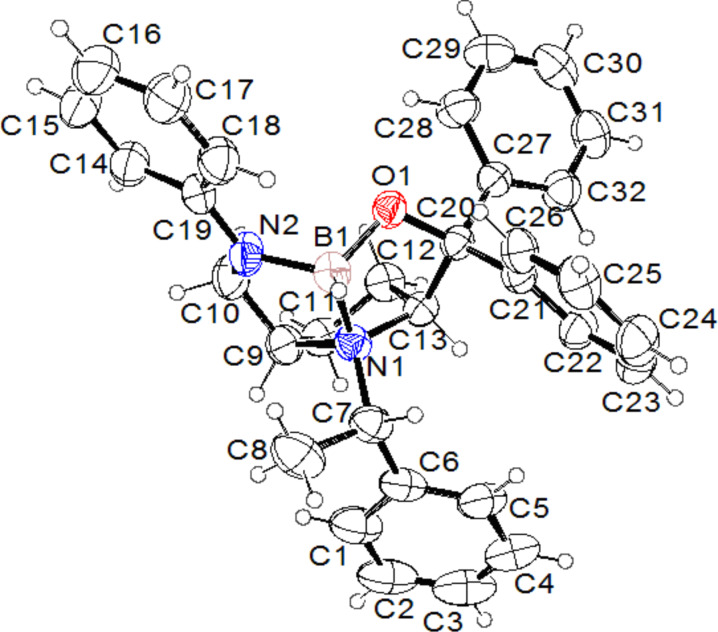
X-ray crystal structure of compound **5a**.

For the reduction of highly hindered amides **4**, the system of LiAlH_4_ in anhydrous THF was found to be useless even upon heating under reflux for 18 h under an inert atmosphere, and no desired diamino-alcohols were obtained after workup. However, **4a** reacted smoothly with BH_3_ to give white crystals after chromatographic purification (Scheme 2). The X-ray single-crystal diffraction analysis established that the reduction product **5a** (Figure 3) was formed with a rigid 10-heteroazaquinane skeleton through an intramolecular Lewis acid–base pair interaction [15–18]. Three five-membered rings are fused to give a very stable bowl-shaped tricyclic system with five stereogenic centers, especially for one chiral nitrogen center and one chiral boron center. The configurations of the stereogenic centers in **5a** are deduced from the configuration of the chiral auxiliary (*S*)-(−)-1-phenylethylamine to be *S*, 3*S*, 6*R*, 9*S* (chiral B atom) and 10*R* (chiral N atom), respectively. This novel tandem reduction/cyclization was made possible by the *cis*-configuration of the starting 2,5-disubstituted pyrrolidine **4a**. Following the same procedure, 10-heteroazaquinane **5b** was obtained in good yield (87%), and the configurations of **5b** were assigned to be *S*, 3*R*, 6*S*, 9*R* (chiral B atom) and 10*S* (chiral N atom), respectively. Compounds **5a** and **5b** are diastereomers.

**Scheme 3:**
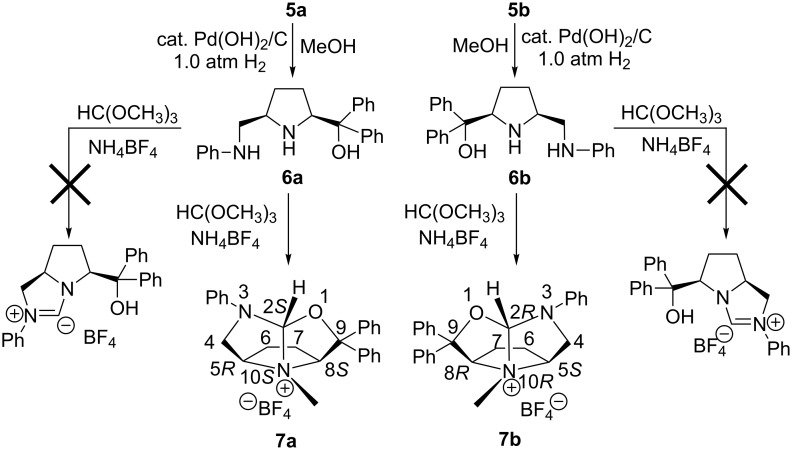
One-pot tandem cyclization of **6** in the presence of HC(OCH_3_)_3_.

In the presence of a catalytic amount of Pd(OH)_2_/C, under 1.0 atm of hydrogen, the above 10-heteroazaquinane derivatives **5** were converted into enantiopure asymmetric *cis*-2,5-disubstituted pyrrolidines **6** in good yields with a diamino-alcohol skeleton. In this process, both *N*-debenzylation and a ring-opening reaction occurred. Diamino-alcohols **6a** and **6b** are enantiomers, and they can serve as precursors for the synthesis of hydroxy *N*-heterocyclic carbenes. Following the reported procedure for the preparation of *N*-heterocyclic carbenes [19–20], the enantiopure pyrrolidine **6b** and NH_4_BF_4_ in HC(OCH_3_)_3_ is heated to 80 °C for 2 h, the light yellow crystals were obtained in good yield after workup (Scheme 3). The same result was obtained by heating the mixture of **6b**, NH_4_BF_4_ and HC(OCH_3_)_3_ in anhydrous toluene under reflux.

**Figure 4:**
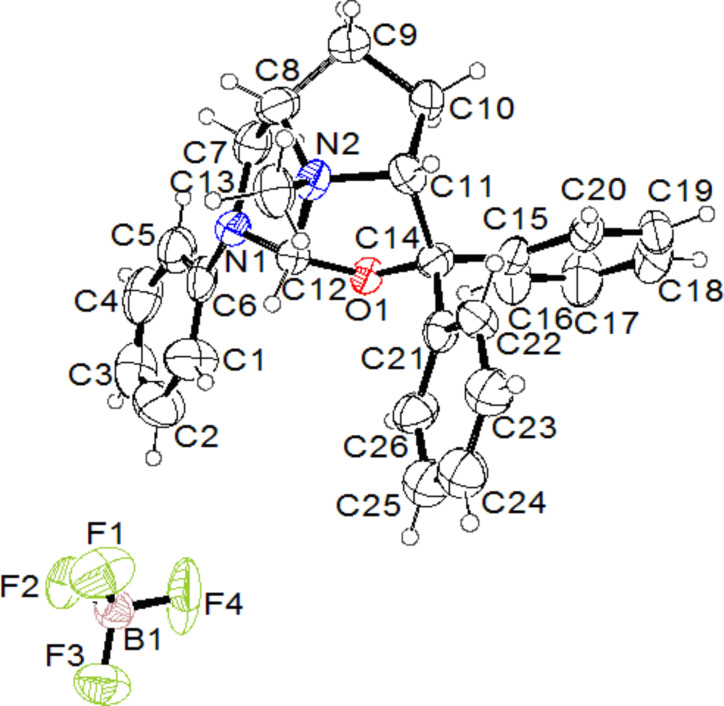
X-ray crystal structure of compound **7b**.

To our delight, a single crystal was grown from CH_2_Cl_2_, suitable for X-ray diffraction analysis. It was found that the ring-closing reaction took place during the heating process following *N*-methylation to provide the rigid 1-oxo-3-aza-10-azaquinane skeleton **7b** as its ammonium salt. Compound **7b** contains four stereogenic centers, and their configurations are assigned to be 2*R*, 5*S*, 8*R* and 10*R* (chiral N atom) based on its starting material **4b** (Figure 4). Actually, HC(OCH_3_)_3_ as an efficient reagent for *C*-, *N*-, and *O*-methylation has been reported [21–24], but the mechanism of these methylations is elusive. The other chiral ammonium salt **7a** was obtained under the same conditions as those for the preparation of **7b**. The chiral ammonium salts **7a** and **7b** were derived from the enantiomers **6a** and **6b**, therefore, **7a** and **7b** are also enantiomers. The configurations of **7a** are assigned to be 2*S*, 5*R*, 8*S* and 10*S* (chiral N atom).

**Figure 5:**
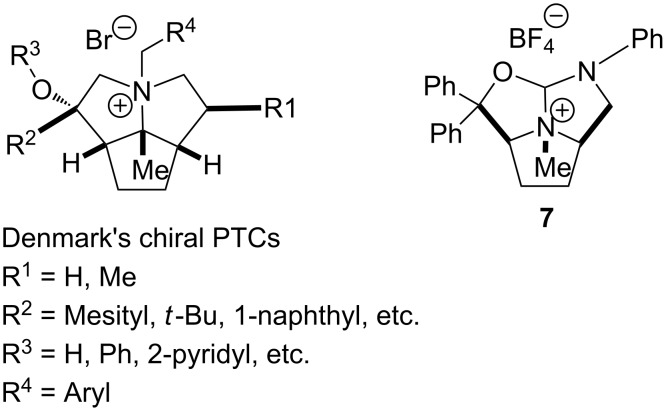
The structural comparison of chiral ammonium salts such as **7** with the chiral PTCs of Denmark et al.

As shown in Figure 4, this 10-heteroazatriquinane **7b** possesses a rigid bowl-like molecular scaffold with a quaternary nitrogen site (N2) at the bottom of the cavity. Recently, Denmark and colleagues [25–26] have synthesized a series of chiral phase-transfer catalysts (chiral PTCs) based on a 2-azatriquinane skeleton (Figure 5), and they have investigated their catalytic activities in asymmetric alkylation reactions for producing enantiomerically enriched amino acids. The synthesis of these quaternary ammonium ions follows a diversity-oriented approach wherein the tandem inter-[4 + 2]/intra-[3 + 2] cycloaddition of nitroalkenes serves as the key transformation. The chiral ammonium salts **7** have a structure similar to the chiral PTCs of Denmark et al. The substituents in the chiral ammonium salts **7** are easily tunable, which could open a route to various chiral PTCs for organic synthesis.

## Conclusion

In summary, we provide here a facile access to chiral 10-heteroazatriquinanes from enantiopure asymmetric *cis*-2,5-disubstituted pyrrolidines through one-pot tandem cyclization reactions, and their configurations are confirmed by X-ray single-crystal diffraction analysis. The applications of these novel 10-heteroazatriquinanes are currently being investigated in our laboratory.

## Supporting Information

File 1 Full experimental details, analytical data and crystallographic information.
