# Supplementary material for: One-pot tandem cyclization of enantiopure asymmetric cis-2,5-disubstituted pyrrolidines: Facile access to chiral 10-heteroazatriquinanes
Source: Beilstein J Org Chem. 2013 Feb 7;9:265–9. doi: 10.3762/bjoc.9.32 (PMC3596118; doi:10.3762/bjoc.9.32)
Supplement: File 1 — Full experimental details, analytical data and crystallographic information. [file Beilstein_J_Org_Chem-09-265-s001.pdf]

Supporting Information

for

One-pot tandem cyclization of enantiopure asymmetric *cis*-2,5-disubstituted pyrrolidines: Facile access to chiral 10-heteroazatriquinanes

Ping-An Wang*[1], Sheng-Yong Zhang[1] and Henri B. Kagan[2]

Address: [1]Department of Medicinal Chemistry, School of Pharmacy, The Fourth Military Medical University, Changle Xilu 17, Xi-An, 710032, P. R. China and [2]Institut de Chimie Moléculaire et des Matériaux d'Orsay (ICMMO-UMR 8182, CNRS), Laboratoire de Catalyse Moléculaire, Université Paris-Sud, 15 rue Georges Clemenceau, 91405 Orsay Cedex, France

Email: Ping-An Wang - ping_an1718@yahoo.com.cn

* Corresponding author

Full experimental details, analytical data and crystallographic information.

A. General

Melting points are uncorrected and expressed in °C. ^{1}H NMR and ^{13}C NMR spectra were measured in $CDCl_3$ or MeOD solution on a Bruker AV-500 spectrometer using TMS as an internal reference. Coupling constant (*J*) values are given in Hz. Multiplicities are designated by the following abbreviations: s, singlet; d, doublet; t, triplet; q, quartet; br, broad; m, multiplet. Optical rotations analyses were performed on a Perkin-Elmer Model 343 Polarimeter. Low- and high-resolution mass spectra were performed on a VG Micromass 7070F Mass Spectrometer with ES ionization (ESI). All commercially available reagents were used as received. Products were purified by flash column chromatography on silica gel purchased from Qingdao Haiyang Chemical Co., Ltd. All reactions involving air- or moisture-sensitive species were performed in oven-dried glassware under inert atmosphere.

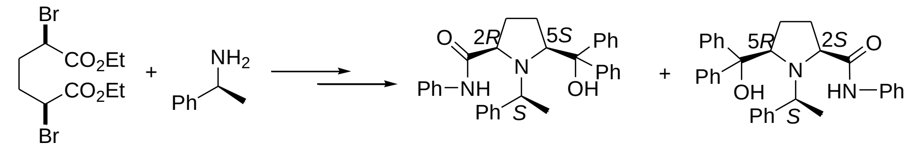

***meso*-** **4a** **4b**

Compounds **4a** and **4b** were synthesized from commercially available starting materials diethyl *meso*-2,5-dibromoadipate and (*S*)-(−)-1-phenylethylamine according to our previous literature procedure [1]. Crystallographic data of **4b** have been deposited with the Cambridge Crystallographic Data Centre (CCDC# 633772). Copies of the data can be obtained free of charge on application to the CCDC, 12 Union Road, Cambridge CB21EZ, UK (fax: (+44)-1223-336-033; e-mail: deposit@ccdc.cam.ac.uk).

Chemical names were generated using ChemDraw Ultra 12.0 (CambridgeSoft).

B. General Procedures

General procedure for reduction of the highly hindered amides 4 by BH_3 in THF

As described in [1], to a solution of compound **4a** or **4b** (7.2 g, 15 mmol) in absolute THF (120 mL) was added dropwise BH_3·THF (30.0 mL, 60 mmol) at rt under an inert atmosphere, and the reaction mixture was stirred for 1.0 h. Then, the reaction mixture was heated under reflux and stirred for 24 h. The reaction was examined by TLC. The mixture was cooled to 0°C and carefully quenched by the addition of methanol (10.0 mL) and water (10.0 mL). The mixture was evaporated under reduced pressure and the residue was dissolved in EtOAc (180 mL). After removal of solid by filtration, the cake was washed with EtOAc (2 × 50 mL). The combined filtrate was washed with water and brine and dried over anhydrous Na_2SO_4. The solvent was evaporated to give a crude product as a white foam. The pure product **5a** or **5b** was obtained by flash column chromatography on silica gel (eluant: hexane/EtOAc). The single crystals of **5a** were obtained by slow evaporation from the mixed solvents of CH_2Cl_2/hexane.

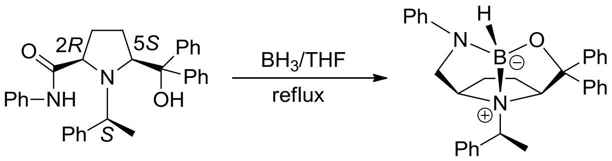

4a **5a**

5a (2a*S*,2a^1*R*,4a*R*,6a*S*)-2,2,6-Triphenyl-2a^1-((*S*)-1-phenylethyl)octahydro-2*H*-1-oxa-2a^1,6-diaza-6a-boracyclopenta[*cd*]pentalen-2a^1-ium-7-uide

R_f (hexane/EtOAc = 4:1): 0.40; Yield: 87%; white powder; m.p. 214–216°C; $[\alpha]_D^{20}$ = +165.4 (c = 1.0, $CHCl_3$);

IR (neat): 3431, 2934, 1366, 1047, 991, 880, 839, 748, 707 cm^{-1};

^1H NMR ($CDCl_3$, 500 MHz): δ 7.90–7.88 (m, 2H), 7.56–6.95 (m, 17H), 6.68 (m, 1H), 4.65–4.63 (t, J = 7.0 Hz, 1H), 4.40–4.36 (m, 1H), 4.03–3.98 (q, J = 7.0 Hz, 1H), 3.86–

3.82 (t, J = 8.5 Hz, 1H), 3.32–3.29 (dd, J = 6.0, 4.0 Hz, 1H), 1.80–1.76 (m, 1H), 1.63–1.50 (m, 5H), 1.46–1.41 (m, 1H);

^{13}C NMR ($CDCl_3$, 125 MHz): δ 149.6, 146.6, 146.3, 138.7, 129.4, 129.2, 129.1, 128.9, 128.1, 127.8, 126.8, 126.6, 126.1, 125.7, 114.8, 113.6, 84.5, 74.5, 65.6, 64.0, 52.4, 30.6, 29.5, 18.7;

LRMS (ESI) *m*/*z* (%) = 472.28 (25.6) [M (^{10}B) + H]$^+$, 473.28 (100) [M (^{11}B) + H]$^+$;

HRMS (ESI) *m*/*z* calcd for $C_{32}H_{34}BN_2O$ ([M + H]$^+$) 473.2764, found 473.2756.

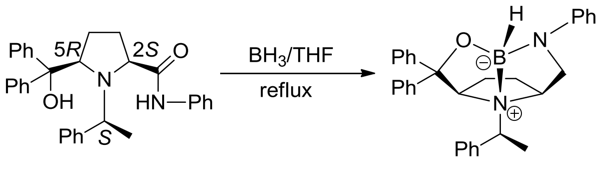

4b **5b**

5b (2a*R*,2a^1*S*,4a*S*,6a*R*)-2,2,6-Triphenyl-2a^1-((*S*)-1-phenylethyl)octahydro-2*H*-1-oxa-2a^1,6-diaza-6a-boracyclopenta[*cd*]pentalen-2a^1-ium-7-uide

R_f (hexane/EtOAc = 3:1): 0.45; Yield: 91%; white powder; m.p. 188–190°C; $[\alpha]_D^{20}$ = −210.5 (c = 1.0, $CHCl_3$);

IR (neat): 3435, 2939, 1366, 1179, 1062, 986, 753, 693 cm^{-1};

^{1}H NMR ($CDCl_3$, 500 MHz): δ 7.83–7.81 (m, 2H), 7.66–7.65 (m, 2H), 7.41–7.13 (m, 13H), 6.84–6.82 (m, 2H), 6.69–6.66 (m, 1H), 4.97–4.95 (m, 1H), 4.15–4.11 (m, 1H), 3.80–3.78 (m, 1H), 3.44–3.39 (m, 1H), 3.32–3.29 (dd, J = 7.5, 2.5 Hz, 1H), 2.09–2.02 (m, 1H), 1.74–1.69 (m, 2H), 1.60–1.57 (m, 1H), 1.38–1.36 (d, J = 7.0 Hz, 3H);

^{13}C NMR ($CDCl_3$, 125 MHz): δ 149.8, 147.5, 146.1, 138.5, 129.2, 128.9, 128.8, 128.7, 128.1, 127.8, 126.4, 126.3, 126.2, 126.1, 114.8, 113.3, 86.1, 71.3, 66.7, 64.7, 49.7, 31.2, 30.6, 19.3;

LRMS (ESI) *m*/*z* (%) = 472.29 (24.4) [M (^{10}B) + H]$^+$, 473.29 (100) [M (^{11}B) + H]$^+$;

HRMS (ESI) *m*/*z* calcd for $C_{32}H_{34}BN_2O$ ([M + H]$^+$) 473.2764, found 473.2757.

General procedure for tandem debenzylation-ring opening of 5

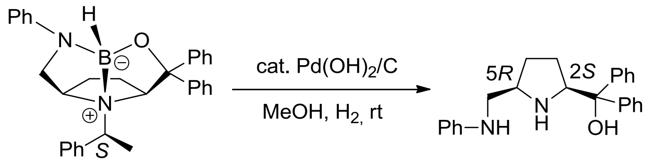

5a **6a**

As described in [1], a solution of compound **5a** or **5b** (5 mmol) in MeOH (50 mL) was hydrogenated (1.0 atm H_2) over 20% $Pd(OH)_2/C$ (700 mg, 1.0 mmol) for 24 h. The air was removed gently by vacuum before introduction of H_2. The catalyst was removed by filtration through Celite and the cake was washed with MeOH (2 × 10 mL). The combined filtrate was evaporated to give crude product as a white foam, which was recrystallized from MeOH–Et_2O (v/v = 1:10). After recrystallization, the pure product **6a** or **6b** was obtained as a white needle crystal.

6a Diphenyl((2*S*,5*R*)-5-((phenylamino)methyl)pyrrolidin-2-yl)methanol

Yield: 81%; white needle crystal; m.p. 199–201°C (dec.); $[\alpha]_D^{20}$ = −39.1 (*c* = 1.0, MeOH);

IR (neat): 3405, 3030, 2942, 1320, 1260, 1067, 756, 699 cm^{-1};

^{1}H NMR (MeOD, 500 MHz): δ 7.59–7.15 (m, 12H), 6.74–6.71 (m, 3H), 5.00–4.98 (m, 1H), 3.89–3.88 (m, 1H), 3.54–3.44 (m, 2H), 2.28–1.97 (m, 4H);

^{13}C NMR ($CDCl_3$, 125 MHz): δ 147.8, 144.0, 143.8, 128.9, 128.5, 128.2, 127.4, 127.3, 125.5, 125.4, 117.8, 112.9, 77.2, 67.2, 60.6, 43.4, 27.6, 25.1;

HRMS (ESI) *m*/*z* calcd for $C_{24}H_{27}N_2O$ ($[M + H]^+$) 359.2123, found 359.2103.

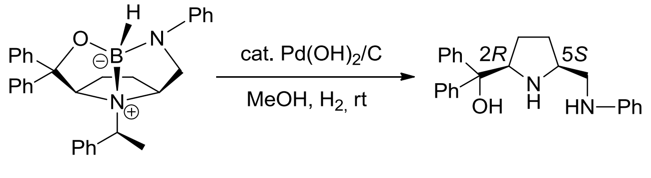

5b **6b**

6b Diphenyl((2*R*,5*S*)-5-((phenylamino)methyl)pyrrolidin-2-yl)methanol

Yield: 78%; white needle crystal; m.p. 236–238°C (dec.); $[\alpha]_D^{20}$ = +38.8 (*c* = 1.0, MeOH);

IR (neat): 3385, 3028, 2933, 1315, 1260, 1086, 758, 707 cm^{-1};

^1H NMR (MeOD, 500 MHz): δ 7.58–7.15 (m, 12H), 6.74–6.70 (m, 3H), 4.97–4.95 (m, 1H), 3.87–3.86 (m, 1H), 3.53–3.44 (m, 2H), 2.28–1.96 (m, 4H);

^{13}C NMR ($CDCl_3$, 125 MHz): δ 147.8, 144.1, 143.8, 128.9, 128.5, 128.2, 127.3, 127.2, 125.5, 125.4, 117.7, 112.7, 77.2, 67.1, 60.5, 43.3, 27.7, 25.0;

HRMS (ESI) *m/z* calcd for $C_{24}H_{27}N_2O$ ($[M + H]^+$) 359.2123, found 359.2144.

General procedure for tandem cyclization-methylation of 6

A mixture of **6a** or **6b** (0.72 g, 2.0 mmol) and NH_4BF_4 (0.43 g, 4.0 mmol) in 7.0 mL of trimethyl orthoformate (65 mmol) was heated under reflux for 8 h under an Ar atmosphere. The reaction was examined by TLC. After the starting material disappeared, the remaining trimethyl orthoformate was evaporated under reduced pressure to give a brown oily residue, which was washed with *n*-hexane (2 × 3.0 mL) and recrystallized from *n*-hexane and CH_2Cl_2 (v/v = 10:1) to give **7a** or **7b** as light yellow crystals. The single crystals of **7b** were obtained by slow evaporation from the mixed solvents of CH_2Cl_2/MeOH.

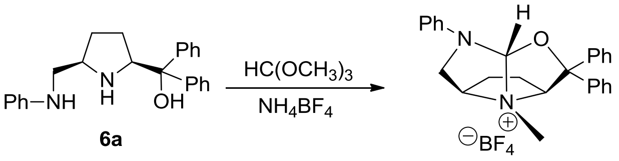

6a **7a**

7a (2a*S*,2a[1]*S*,4a*R*,6a*S*)-2a[1]-Methyl-2,2,6-triphenyloctahydro-2*H*-1-oxa-2a[1],6-diazacyclopenta[*cd*]pentalen-2a[1]-ium tetrafluoroborate

Yield: 82%; light yellow crystal; m.p. 212–214°C (dec.); $[\alpha]_D^{20}$ = +23.6 (*c* = 1.0, $CHCl_3$);

^1H NMR ($CDCl_3$, 500 MHz): δ 7.88–7.86 (m, 2H), 7.56–6.99 (m, 13H), 6.14 (s, 1H), 5.83–5.80 (t, *J* = 8.0 Hz, 1H), 4.69–4.66 (t, *J* = 8.0 Hz, 1H), 4.42–4.38 (t, *J* = 8.0 Hz,

1H), 3.64–3.60 (t, *J* = 9.0 Hz, 1H), 3.32 (s, 3H, CH_3), 2.83–2.74 (m, 1H), 2.55–2.53 (m, 1H), 2.12–2.11 (m, 1H), 1.91–1.83 (m, 1H);

^{13}C NMR ($CDCl_3$, 125 MHz): δ 147.4, 139.6, 139.1, 130.1, 130.0, 129.3, 128.9, 128.1, 125.2, 124.4, 122.6, 116.1, 108.5, 89.9, 79.6, 79.3, 51.1, 49.7, 30.6, 27.4;

HRMS (ESI) *m*/*z* calcd for $C_{26}H_{27}N_2O$ ([M – BF_4]$^+$) 383.2123, found 383.2119.

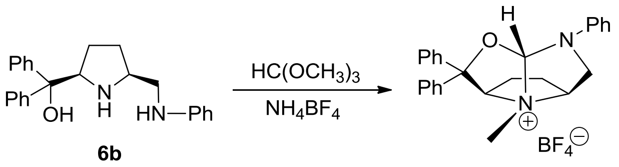

7b (2a*R*,2a^1*R*,4a*S*,6a*R*)-2a^1-Methyl-2,2,6-triphenyloctahydro-2*H*-1-oxa-2a^1,6-diazacyclopenta[*cd*]pentalen-2a^1-ium

Yield: 85%; light yellow crystal; m.p. 211–213°C (dec.); $[\alpha]_D^{20}$ = −24.3 (*c* = 1.0, $CHCl_3$);

^{1}H NMR ($CDCl_3$, 500 MHz): δ 7.87–7.86 (m, 2H), 7.55–7.00 (m, 13H), 6.14 (s, 1H), 5.80–5.77 (t, *J* = 8.5 Hz, 1H), 4.72–4.68 (t, *J* = 7.5 Hz, 1H), 4.44–4.40 (m, 1H), 3.62–3.58 (m, 1H), 3.29 (s, 3H, CH_3), 2.75–2.70 (m, 1H), 2.52–2.49 (m, 1H), 2.15–2.10 (m, 1H), 1.92–1.85 (m, 1H);

^{13}C NMR ($CDCl_3$, 125 MHz): δ 147.4, 139.6, 139.0, 130.1, 130.0, 129.9, 129.2, 128.9, 128.1, 125.2, 124.4, 122.6, 116.1, 108.5, 89.9, 79.6, 79.3, 51.1, 49.7, 30.6, 27.4;

HRMS (ESI) *m*/*z* calcd for $C_{26}H_{27}N_2O$ ([M – BF_4]$^+$) 383.2123, found 383.2117.

X-ray crystal structure data for compounds 5a and 7b

1. X-ray crystallographic data for compound 5a

Crystals of compound **5a** suitable for X-ray analysis were obtained by slow evaporation from the mixed solvents of CH_2Cl_2/hexane. Crystallographic data have been deposited with the Cambridge Crystallographic Data Centre (CCDC# 891107). Copies of the data can be obtained free of charge on application to the CCDC, 12 Union Road, Cambridge CB21EZ, UK (fax: (+44)-1223-336-033; e-mail: deposit@ccdc.cam.ac.uk).

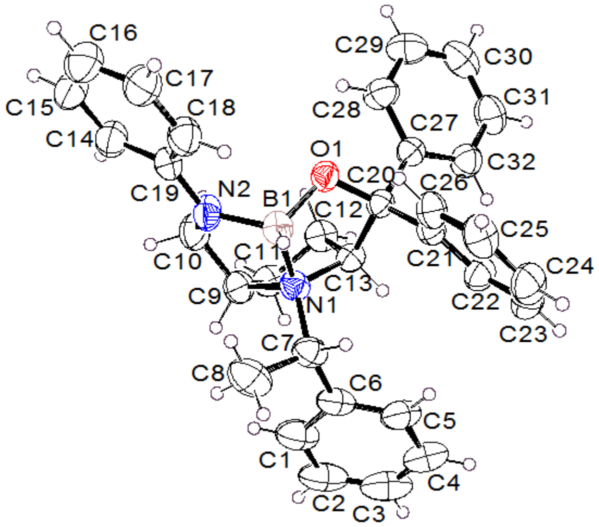

Table S1: Crystal data and structure refinement for compound **5a**.

Identification code	compound 5a
Empirical formula	C32 H33 B N2 O
Formula weight	472.41
Temperature	296(2) K
Wavelength	0.71073 Å
Crystal colour	colourless
Crystal description	block

Crystal system	Orthorhombic
Space group	P2(1)2(1)2(1)
Unit cell dimensions	a = 9.0271(16) Å α = 90°.
	b = 14.669(3) Å β = 90°.
	c = 19.643(3) Å γ = 90°.
Volume	2601.0(8) $Å^3$
Z	4
Calculated density	1.206 Mg/m^3
Absorption coefficient	0.072 mm^{-1}
F(000)	1008
Crystal size	0.38 x 0.29 x 0.17 mm
Theta range for data collection	1.73 to 25.10°
Limiting indices	-10<=h<=10, -17<=k<=12, -22<=l<=23
Reflections collected	13137
Independent reflections	4635 [R(int) = 0.0317]
Completeness to theta = 25.10	99.8 %
Absorption correction	constr
Max. and min. transmission	0.9880 and 0.9735
Refinement method	Full-matrix least-squares on F^2
Data / restraints / parameters	4635 / 0 / 327
Goodness-of-fit on F^2	0.990
Final R indices [I>2sigma(I)]	R1 = 0.0363, wR2 = 0.0793
R indices (all data)	R1 = 0.0641, wR2 = 0.0855
Absolute structure parameter	0.0(14)
Extinction coefficient	0.0085(8)
Largest diff. peak and hole	0.117 and -0.120 e.$Å^{-3}$

Table 2: Atomic coordinates (x 10^4) and equivalent isotropic displacement parameters ($Å^2$ x 10^3) for compound **5a**. U(eq) is defined as one third of the trace of the orthogonalized U^{ij} tensor.

	x	y	z	U(eq)
N(1)	7081(2)	5374(1)	2386(1)	58(1)
N(2)	5005(2)	6240(1)	1929(1)	68(1)
O(1)	6271(1)	5057(1)	1231(1)	56(1)
B(1)	6435(3)	5843(2)	1685(1)	60(1)
C(1)	8905(3)	5397(2)	3877(1)	97(1)
C(2)	9676(4)	4923(2)	4382(1)	106(1)
C(3)	10849(4)	4382(2)	4204(2)	113(1)
C(4)	11292(3)	4317(2)	3551(2)	101(1)
C(5)	10528(3)	4801(2)	3052(1)	85(1)
C(6)	9318(3)	5330(2)	3196(1)	73(1)
C(7)	8526(2)	5828(2)	2622(1)	67(1)
C(8)	8276(3)	6836(2)	2769(1)	102(1)
C(9)	5831(2)	5419(2)	2906(1)	67(1)
C(10)	4474(2)	5778(2)	2533(1)	76(1)
C(11)	5664(2)	4453(2)	3160(1)	73(1)
C(12)	5931(2)	3902(1)	2524(1)	66(1)
C(13)	7241(2)	4366(1)	2198(1)	54(1)
C(14)	2535(3)	6845(2)	1707(1)	79(1)
C(15)	1600(3)	7339(2)	1292(2)	96(1)
C(16)	2064(4)	7698(2)	693(2)	100(1)

C(17)	3506(3)	7548(2)	496(1)	91(1)
C(18)	4474(3)	7060(2)	902(1)	79(1)
C(19)	4008(2)	6700(1)	1523(1)	64(1)
C(20)	7270(2)	4354(1)	1404(1)	51(1)
C(21)	8822(2)	4547(1)	1120(1)	53(1)
C(22)	10066(2)	4099(1)	1374(1)	67(1)
C(23)	11464(2)	4278(2)	1131(1)	80(1)
C(24)	11650(3)	4913(2)	627(1)	86(1)
C(25)	10438(3)	5344(2)	353(1)	79(1)
C(26)	9036(2)	5162(1)	602(1)	64(1)
C(27)	6670(2)	3455(1)	1118(1)	54(1)
C(28)	5292(3)	3432(2)	816(1)	77(1)
C(29)	4721(3)	2633(2)	549(1)	94(1)
C(30)	5509(4)	1849(2)	578(1)	91(1)
C(31)	6866(3)	1844(2)	878(1)	86(1)
C(32)	7442(2)	2648(1)	1149(1)	74(1)

2. X-ray crystallographic data for compound 7b

Crystals of compound **7b** suitable for X-ray analysis were obtained by slow evaporation from the mixed solvents of CH_2Cl_2/MeOH. Crystallographic data have been deposited with the Cambridge Crystallographic Data Centre (CCDC# 891108). Copies of the data can be obtained free of charge on application to the CCDC, 12 Union Road, Cambridge CB21EZ, UK (fax: (+44)-1223-336-033; e-mail: deposit@ccdc.cam.ac.uk).

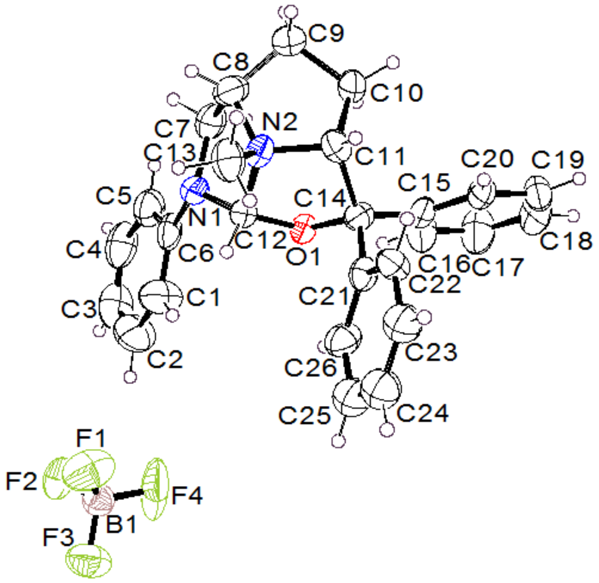

Table S2: Crystal data and structure refinement for compound **7b**.

Identification code	compound **7b**
Empirical formula	C26 H27 B F4 N2 O
Formula weight	470.31
Temperature	296(2) K
Wavelength	0.71073 Å
Crystal colour	colourless
Crystal description	block
Crystal system	Monoclinic

Space group	P2(1)
Unit cell dimensions	a = 8.887(4) Å α = 90°.
	b = 10.259(4) Å β = 101.714(7)°.
	c = 13.390(6) Å γ = 90°.
Volume	1195.4(9) $Å^3$
Z	2
Calculated density	1.307 Mg/m^3
Absorption coefficient	0.100 mm^{-1}
F(000)	492
Crystal size	0.33 x 0.18 x 0.11 mm
Theta range for data collection	1.55 to 25.10 deg.
Limiting indices	-10<=h<=10, -6<=k<=12, -15<=l<=15
Reflections collected	6121
Independent reflections	3281 [R(int) = 0.0691]
Completeness to theta = 25.10	100.0 %
Absorption correction	constr
Max. and min. transmission	0.9891 and 0.9679
Refinement method	Full-matrix least-squares on F^2
Data / restraints / parameters	3281 / 1 / 288
Goodness-of-fit on F^2	0.960
Final R indices [I>2sigma(I)]	R1 = 0.0617, wR2 = 0.1234
R indices (all data)	R1 = 0.1550, wR2 = 0.1654
Absolute structure parameter	2(2)
Largest diff. peak and hole	0.239 and -0.240 e.$Å^{-3}$

Table 2: Atomic coordinates (x 10^4) and equivalent isotropic displacement parameters ($Å^2$ x 10^3) for compound **7b**. U(eq) is defined as one third of the trace of the orthogonalized U^{ij} tensor.

	x	y	z	U(eq)
N(1)	4373(7)	9436(7)	3062(5)	64(2)
O(1)	1863(5)	10141(5)	3066(3)	48(1)
C(1)	4814(11)	9020(12)	4865(8)	95(4)
C(2)	5656(13)	9245(14)	5821(8)	113(5)
C(3)	6889(13)	10073(14)	5980(9)	103(4)
C(4)	7305(12)	10693(12)	5197(11)	99(4)
C(5)	6419(11)	10508(11)	4236(8)	85(3)
C(6)	5202(10)	9719(11)	4070(7)	71(3)
C(7)	4645(8)	10232(10)	2197(7)	71(3)
C(8)	3661(10)	9532(10)	1282(7)	72(3)
C(9)	2826(9)	10383(10)	415(6)	80(3)
C(10)	1426(8)	10897(9)	816(6)	61(2)
C(11)	930(9)	9703(9)	1363(6)	58(2)
N(2)	2370(7)	8927(7)	1716(5)	56(2)
C(12)	2810(8)	9138(8)	2881(6)	51(2)
C(13)	2153(10)	7474(9)	1479(7)	75(3)
C(14)	382(8)	10029(10)	2372(5)	54(2)
C(15)	-463(9)	11327(9)	2344(7)	52(2)
C(16)	43(11)	12266(11)	3022(7)	81(3)
C(17)	-795(12)	13434(13)	3039(8)	108(4)

C(18)	-2158(12)	13560(10)	2352(9)	80(3)
C(19)	-2622(10)	12612(11)	1655(7)	75(3)
C(20)	-1812(9)	11466(9)	1645(6)	55(2)
C(21)	-535(8)	8934(9)	2729(6)	53(2)
C(22)	-1641(10)	8233(9)	2052(7)	67(3)
C(23)	-2557(11)	7296(10)	2405(7)	80(3)
C(24)	-2351(11)	7064(10)	3415(7)	86(3)
C(25)	-1296(11)	7706(11)	4091(7)	83(3)
C(26)	-358(9)	8659(10)	3764(6)	74(3)
B(1)	6500(14)	9336(14)	9140(10)	74(3)
F(1)	6276(7)	8613(8)	8276(4)	139(3)
F(2)	8024(5)	9688(6)	9398(4)	92(2)
F(3)	6105(6)	8590(7)	9869(4)	121(2)
F(4)	5630(6)	10379(7)	8963(8)	204(5)

References:

[1] Wang, P.-A.; Xu, Z.-S.; Chen, C.-F.; Gao, X.-G.; Sun, X.-L.; Zhang, S.-Y. *Chirality* **2007,** *19,* 581–588. doi:10.1002/chir.20424

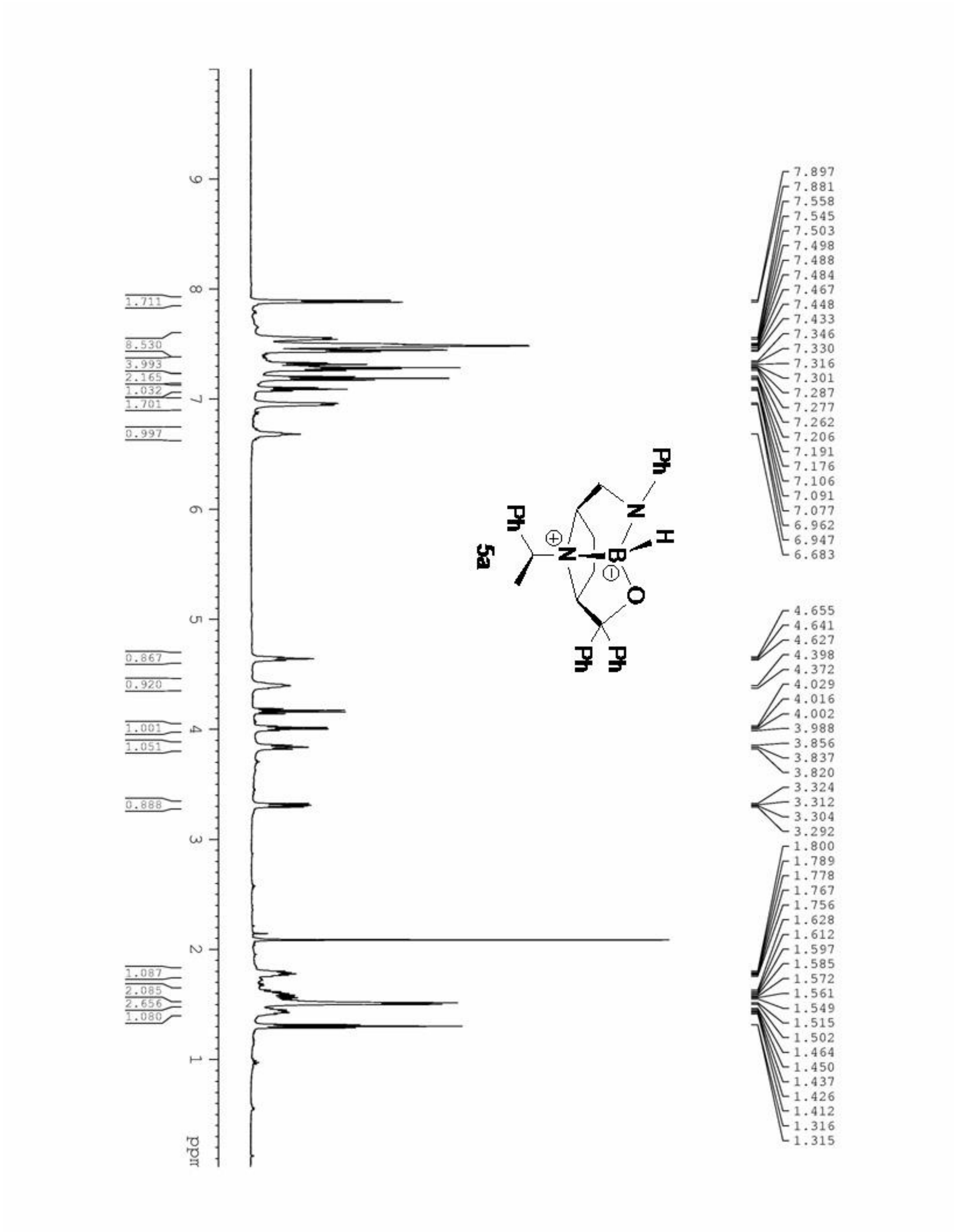
5a
ppm
7.897
7.881
7.558
7.545
7.503
7.498
7.488
7.484
7.467
7.448
7.433
7.346
7.330
7.316
7.301
7.287
7.277
7.262
7.206
7.191
7.176
7.106
7.091
7.077
6.962
6.947
6.683
4.655
4.641
4.627
4.398
4.372
4.029
4.016
4.002
3.988
3.856
3.837
3.820
3.324
3.312
3.304
3.292
1.800
1.789
1.778
1.767
1.756
1.628
1.612
1.597
1.585
1.572
1.561
1.549
1.515
1.502
1.464
1.450
1.437
1.426
1.412
1.316
1.315

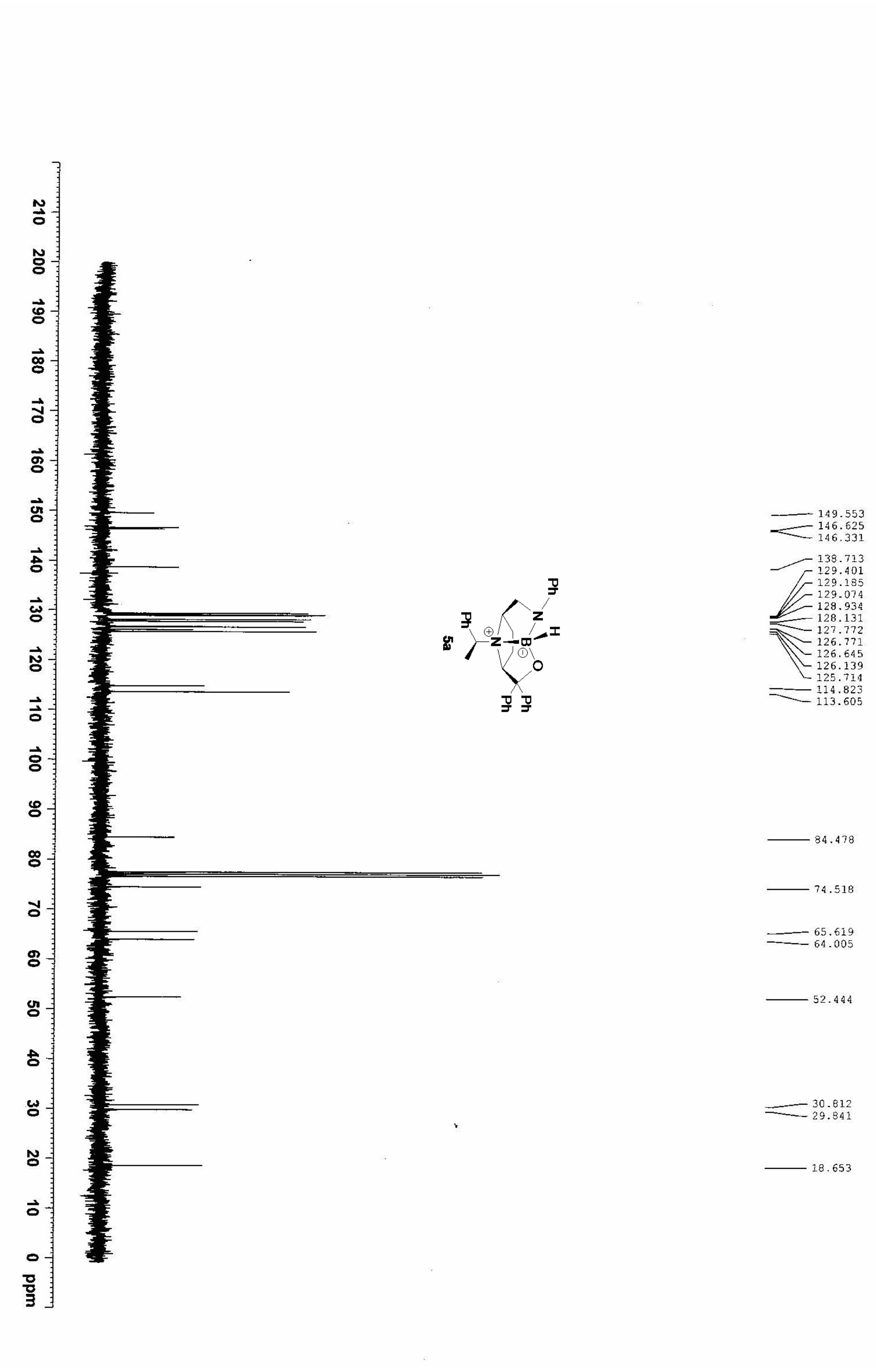
149.553
146.625
146.331
138.713
129.401
129.185
129.074
128.934
128.131
127.772
126.771
126.645
126.139
125.714
114.823
113.605
84.478
74.518
65.619
64.005
52.444
30.812
29.841
18.653
5a
ppm

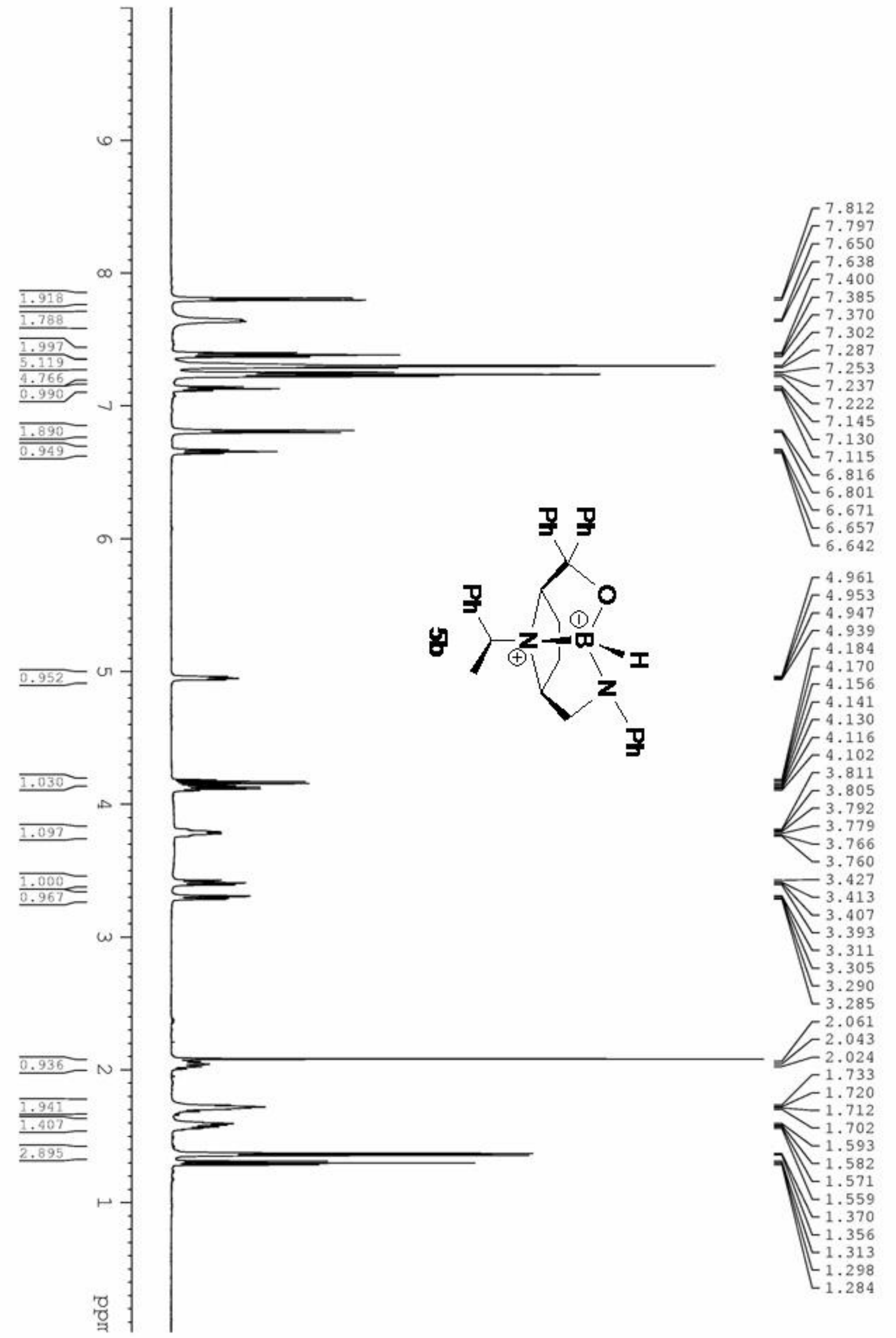
1.918
1.788
1.997
5.119
4.766
0.990
1.890
0.949
0.952
1.030
1.097
1.000
0.967
0.936
1.941
1.407
2.895
9
8
7
6
5
4
3
2
1
ppm
7.812
7.797
7.650
7.638
7.400
7.385
7.370
7.302
7.287
7.253
7.237
7.222
7.145
7.130
7.115
6.816
6.801
6.671
6.657
6.642
4.961
4.953
4.947
4.939
4.184
4.170
4.156
4.141
4.130
4.116
4.102
3.811
3.805
3.792
3.779
3.766
3.760
3.427
3.413
3.407
3.393
3.311
3.305
3.290
3.285
2.061
2.043
2.024
1.733
1.720
1.712
1.702
1.593
1.582
1.571
1.559
1.370
1.356
1.313
1.298
1.284
Ph
Ph
O
B
H
N
Ph
N
Ph
5b

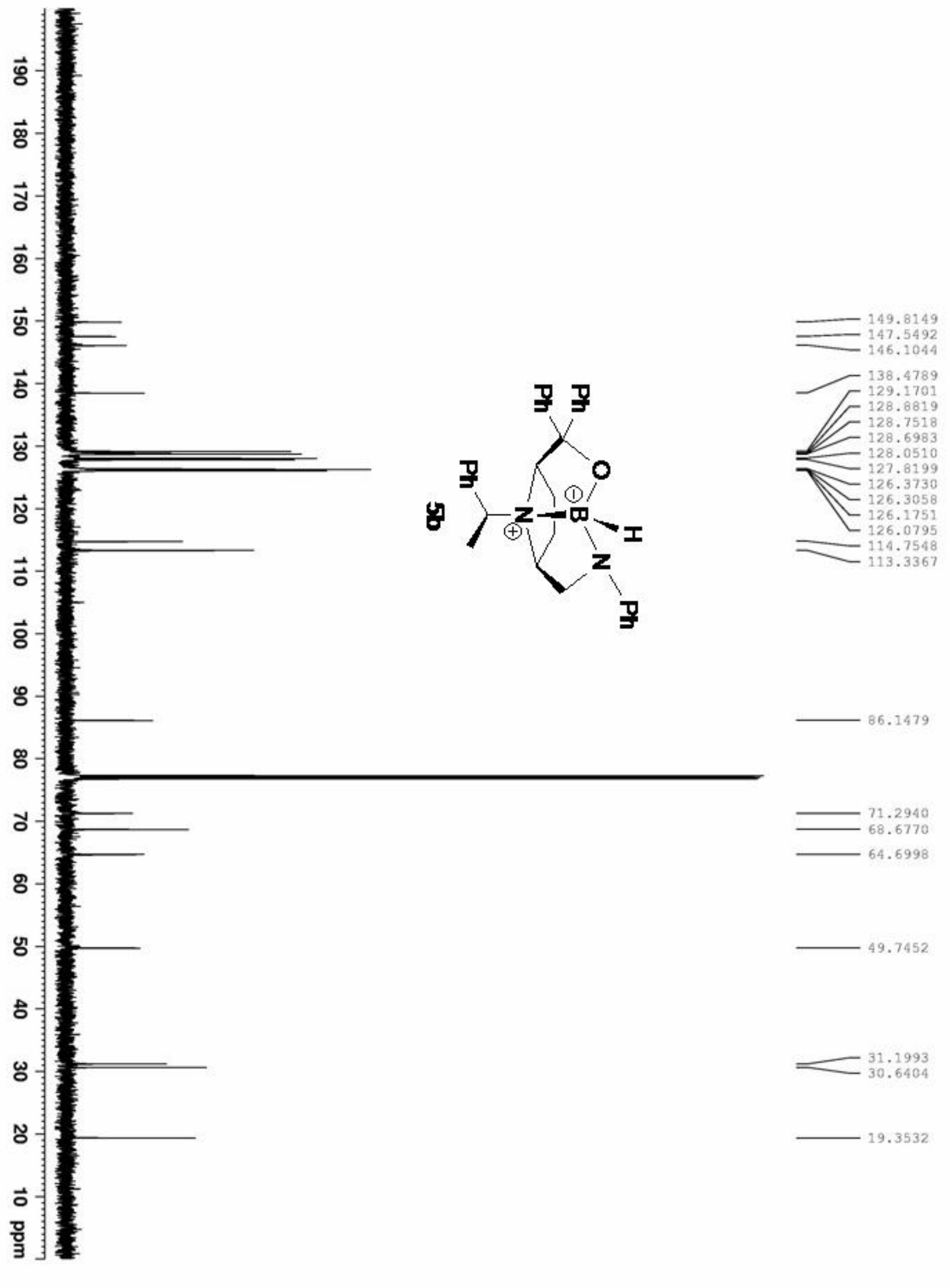

149.8149
147.5492
146.1044
138.4789
129.1701
128.8819
128.7518
128.6983
128.0510
127.8199
126.3730
126.3058
126.1751
126.0795
114.7548
113.3367
86.1479
71.2940
68.6770
64.6998
49.7452
31.1993
30.6404
19.3532
ppm
5b

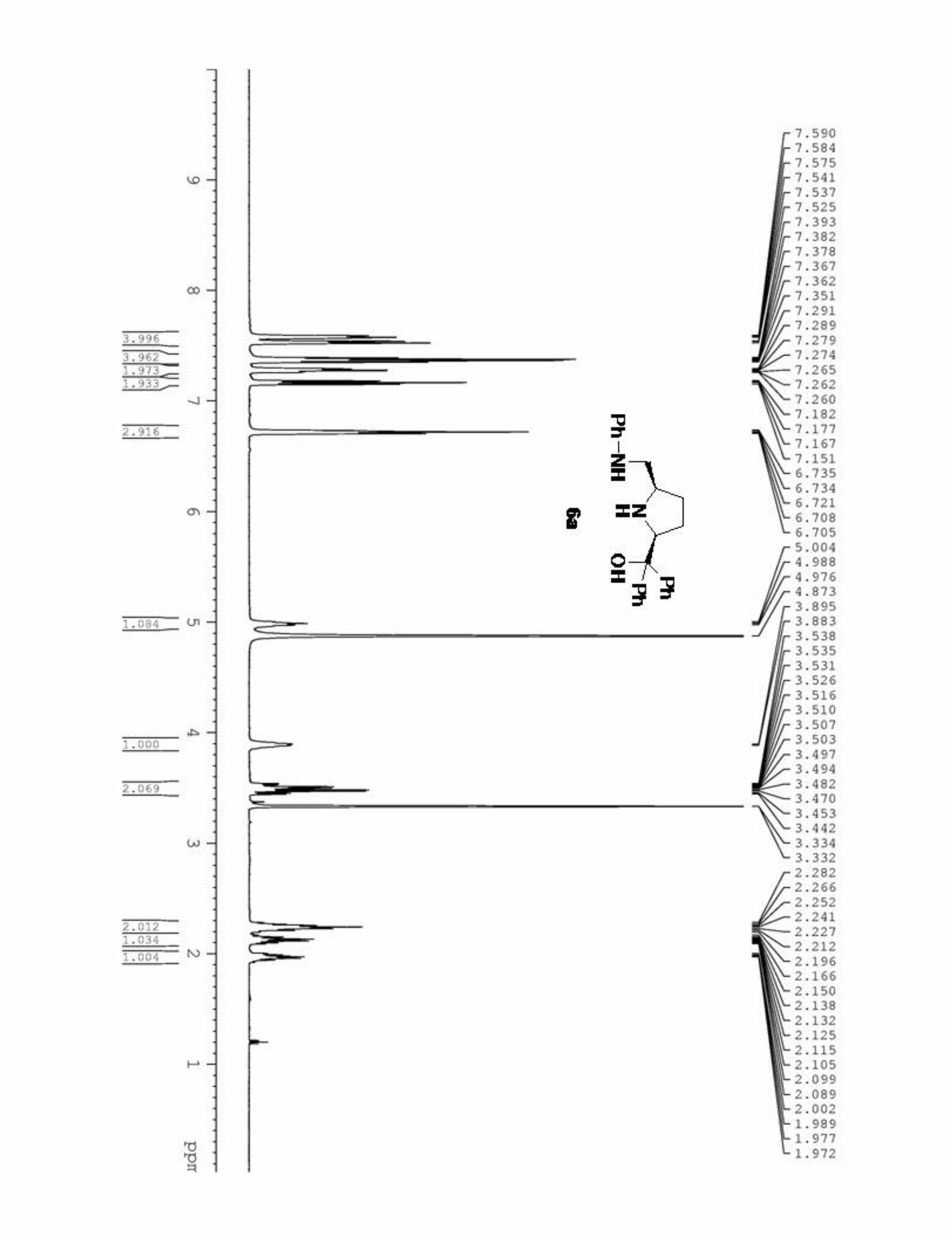

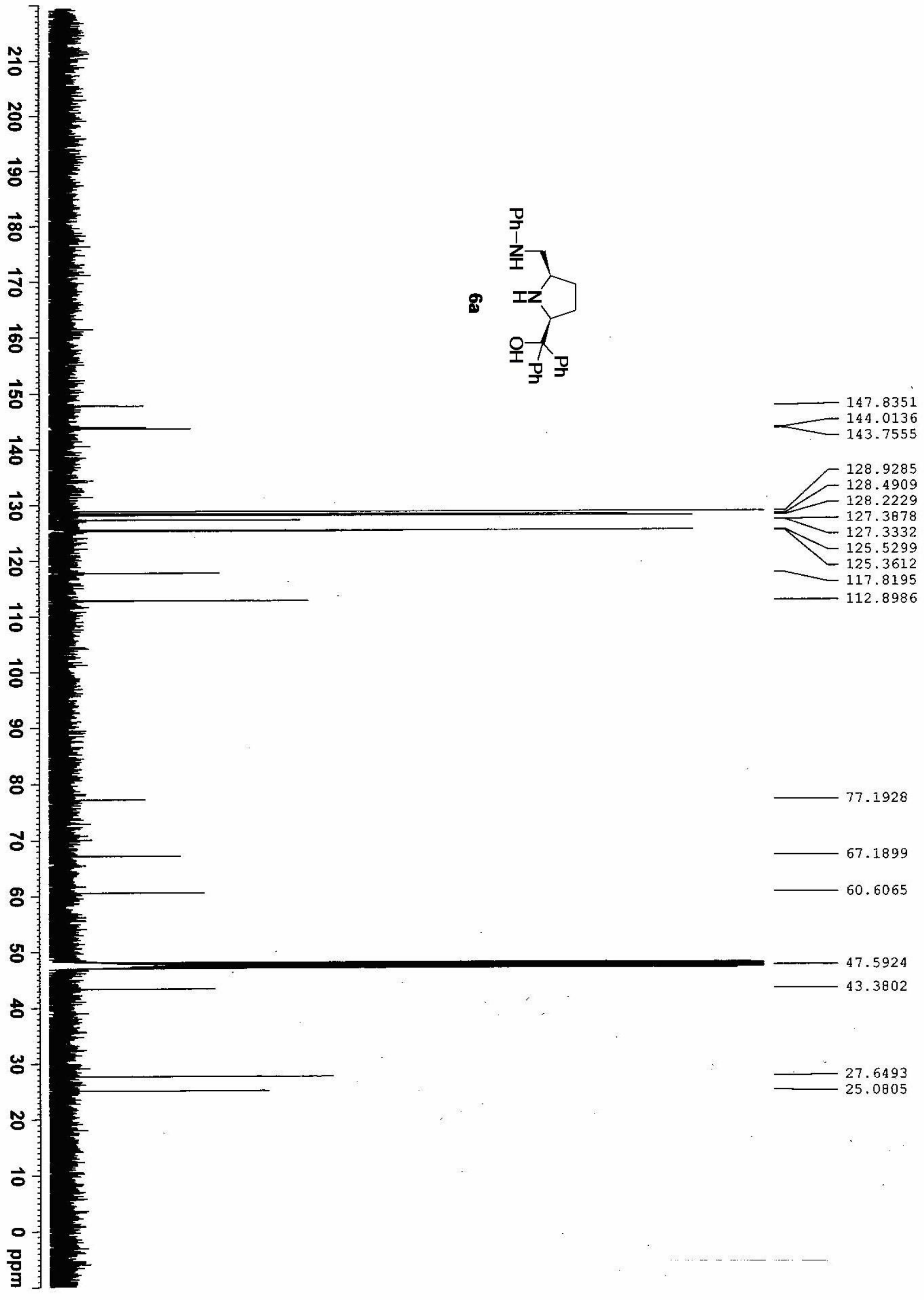
147.8351
144.0136
143.7555
128.9285
128.4909
128.2229
127.3878
127.3332
125.5299
125.3612
117.8195
112.8986
77.1928
67.1899
60.6065
47.5924
43.3802
27.6493
25.0805
6a
ppm

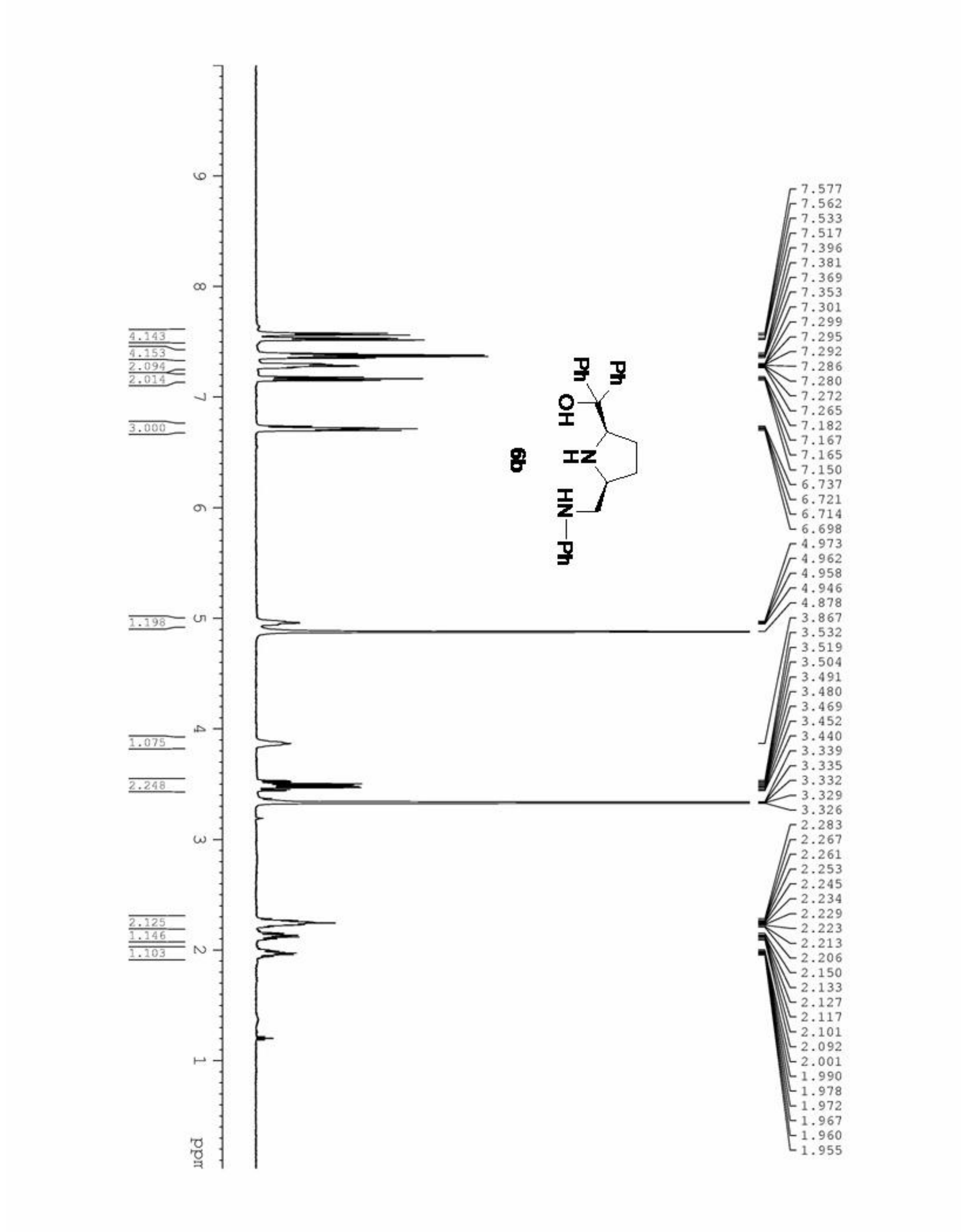
Ph
Ph
OH
N
H
HN—Ph
6b
4.143
4.153
2.094
2.014
3.000
1.198
1.075
2.248
2.125
1.146
1.103
7.577
7.562
7.533
7.517
7.396
7.381
7.369
7.353
7.301
7.299
7.295
7.292
7.286
7.280
7.272
7.265
7.182
7.167
7.165
7.150
6.737
6.721
6.714
6.698
4.973
4.962
4.958
4.946
4.878
3.867
3.532
3.519
3.504
3.491
3.480
3.469
3.452
3.440
3.339
3.335
3.332
3.329
3.326
2.283
2.267
2.261
2.253
2.245
2.234
2.229
2.223
2.213
2.206
2.150
2.133
2.127
2.117
2.101
2.092
2.001
1.990
1.978
1.972
1.967
1.960
1.955
ppm

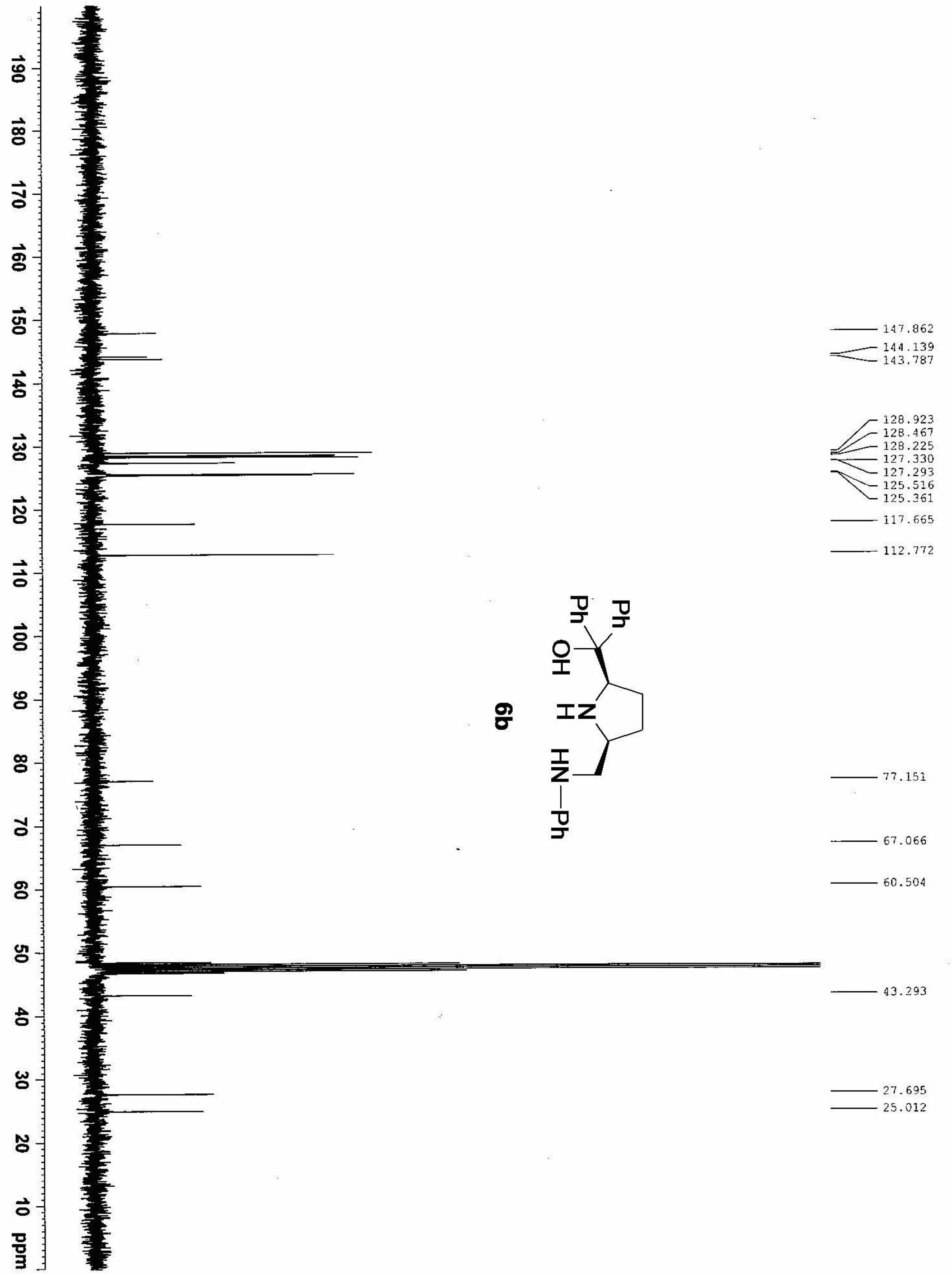
147.862
144.139
143.787
128.923
128.467
128.225
127.330
127.293
125.516
125.361
117.665
112.772
77.151
67.066
60.504
43.293
27.695
25.012
6b
ppm

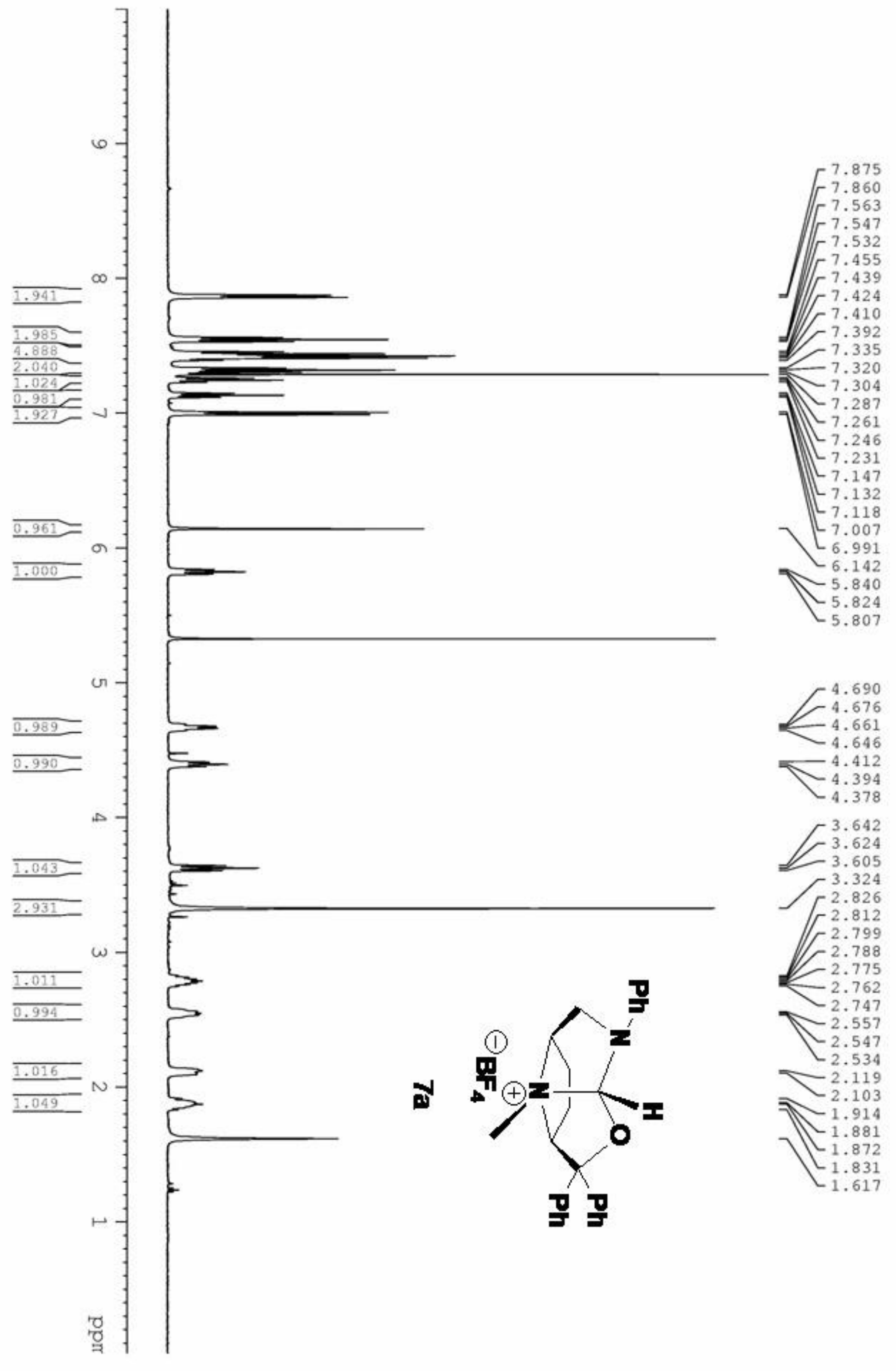
7.875
7.860
7.563
7.547
7.532
7.455
7.439
7.424
7.410
7.392
7.335
7.320
7.304
7.287
7.261
7.246
7.231
7.147
7.132
7.118
7.007
6.991
6.142
5.840
5.824
5.807
4.690
4.676
4.661
4.646
4.412
4.394
4.378
3.642
3.624
3.605
3.324
2.826
2.812
2.799
2.788
2.775
2.762
2.747
2.557
2.547
2.534
2.119
2.103
1.914
1.881
1.872
1.831
1.617
1.941
1.985
4.888
2.040
1.024
0.981
1.927
0.961
1.000
0.989
0.990
1.043
2.931
1.011
0.994
1.016
1.049
ppm
7a

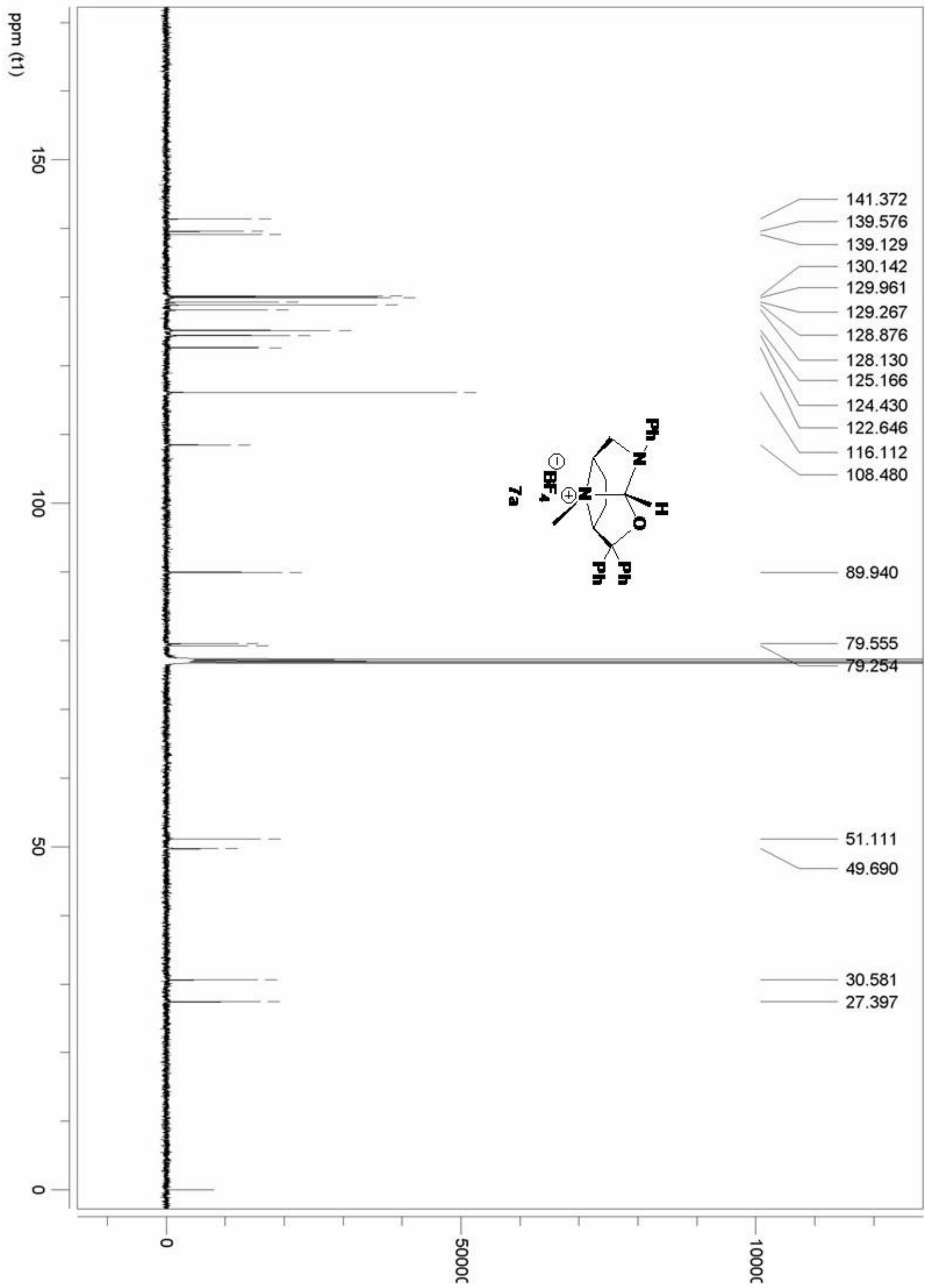
ppm (t1)
150
100
50
0
0
5000
141.372
139.576
139.129
130.142
129.961
129.267
128.876
128.130
125.166
124.430
122.646
116.112
108.480
89.940
79.555
79.254
51.111
49.690
30.581
27.397
Ph
N
H
O
Ph
Ph
N
BF4
7a

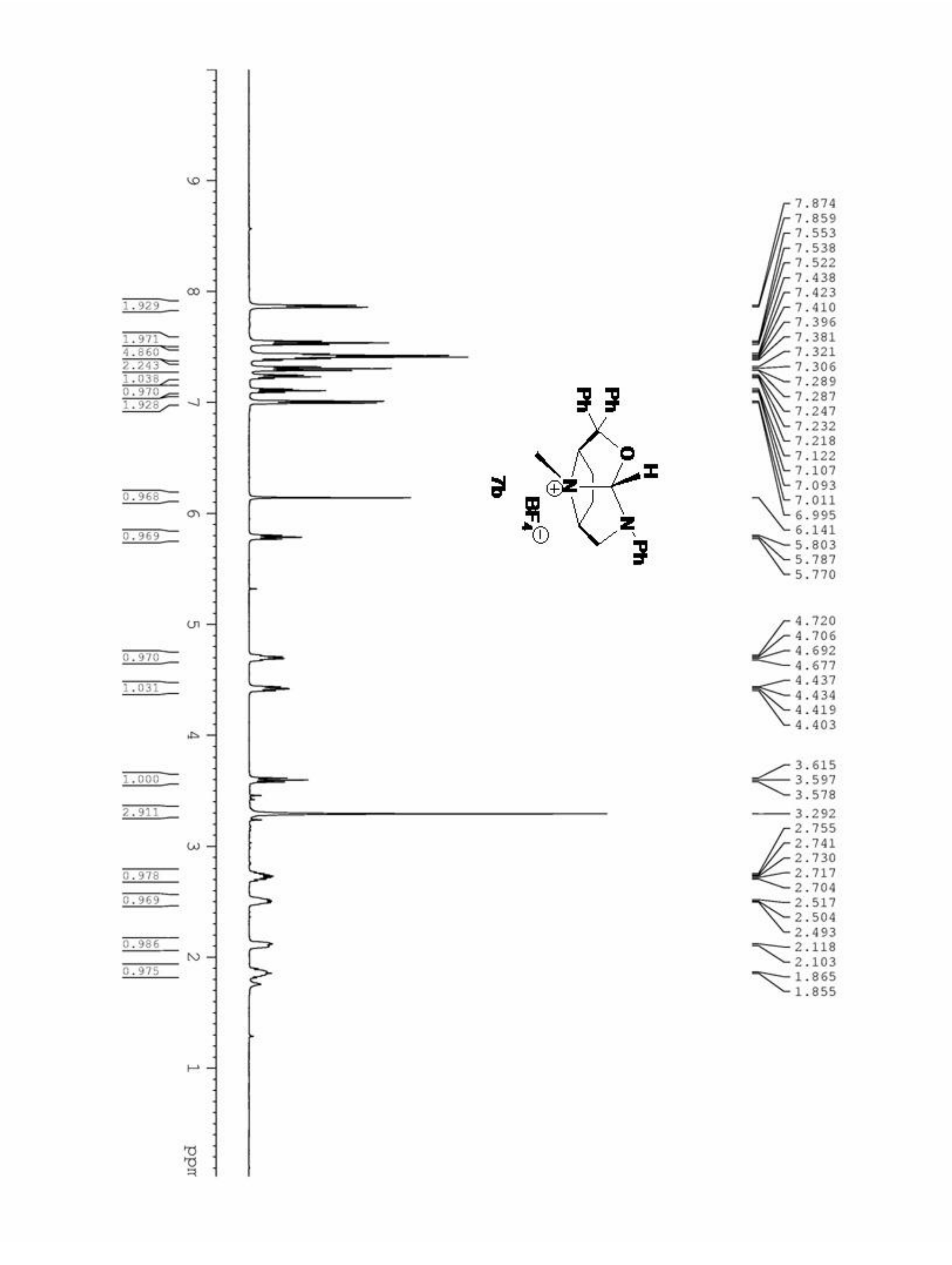
7.874
7.859
7.553
7.538
7.522
7.438
7.423
7.410
7.396
7.381
7.321
7.306
7.289
7.287
7.247
7.232
7.218
7.122
7.107
7.093
7.011
6.995
6.141
5.803
5.787
5.770
4.720
4.706
4.692
4.677
4.437
4.434
4.419
4.403
3.615
3.597
3.578
3.292
2.755
2.741
2.730
2.717
2.704
2.517
2.504
2.493
2.118
2.103
1.865
1.855
1.929
1.971
4.860
2.243
1.038
0.970
1.928
0.968
0.969
0.970
1.031
1.000
2.911
0.978
0.969
0.986
0.975
9
8
7
6
5
4
3
2
1
ppm
Ph
Ph
O
H
N
N
Ph
BF_4
7b

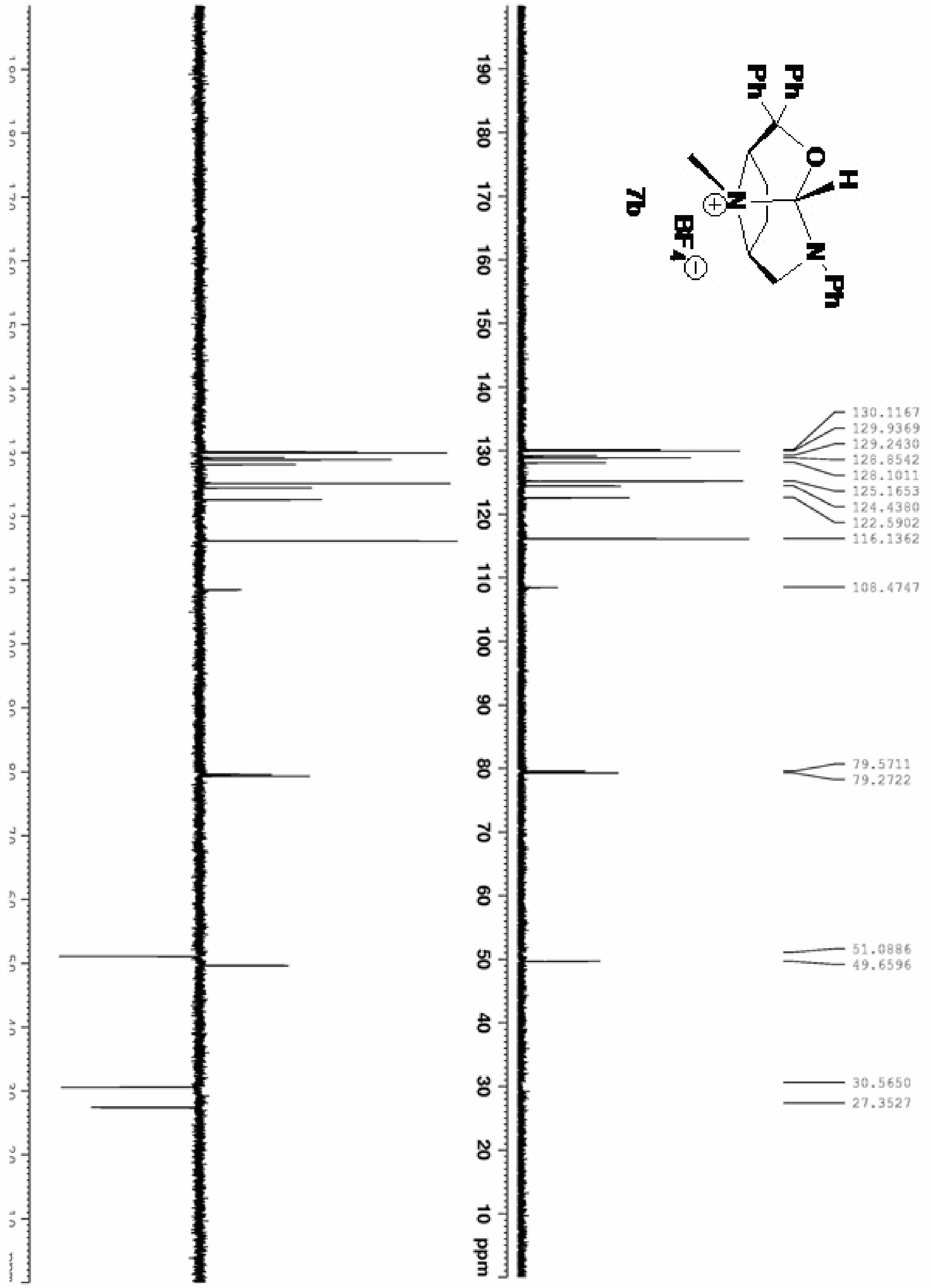
Ph
Ph
O
H
N
N
Ph
BF4
7b
130.1167
129.9369
129.2430
128.8542
128.1011
125.1653
124.4380
122.5902
116.1362
108.4747
79.5711
79.2722
51.0886
49.6596
30.5650
27.3527
190
180
170
160
150
140
130
120
110
100
90
80
70
60
50
40
30
20
10 ppm
